# The New York Institute of Stomatology

**Published:** 1899-06

**Authors:** 

**Affiliations:** The New York Institute of Stomatology


### THE NEW YORK INSTITUTE OF STOMATOLOGY.

A REGULAR meeting of the Institute was held on Tuesday evening March 7, 1899, at the office of Dr. S. H. McNaughton, 63 West Forty-ninth Street, the President, Dr. E. A. Bogue, in the chair.

The minutes of the previous meeting were read and approved.

### COMMUNICATIONS ON THEORY AND PRACTICE.

*The President.*—The President of the National Dental Association (Dr. H. J. Burkhart), in writing to me recently, said that he proposed to urge upon the association the propriety of accepting delegates from such bodies as those we represent, although at present the National Association is composed of delegates from the State societies only, and he has suggested, both to Dr. Allan and myself, that those of us belonging to State societies should cause ourselves to be elected delegates to the National Association, in order that our influence may be used to that end, and he urges that gentlemen of standing and influence should exert that influence in the National Association for the bodies at large.

Before proceeding with the work of the evening are there any questions on theory and practice?

*Dr. W. St. George Elliott.*—I will pass around among the gentlemen some German drills for the pulp-canals. They are said to be excellent, well tempered, and are exceedingly popular on the other side.

*Dr. G. E. Rice.*—I would like to ask the gentleman present their opinion as to the relative desirability of correcting prolongated lower maxillary jaws at an exceedingly early age, or whether it is better to wait until after the permanent teeth have developed?

*Dr. G. S. Allan.*—I have never attempted to move but one child's lower jaw at an early age, and then I had to wait until the permanent teeth had developed. I think I was not very successful.

*Dr. Rice.*—I should like to ask if it is done by moving the teeth forward or moving the lower jaw backward?

*Dr. A. G. Weed.*—There is no doubt whatever that the lower jaw can be moved back with success. The apparatus consists of two rubber bands, and has been used nearly a quarter of a century. I have never used it in a very young child, not below fourteen. My general opinion is that at that age it is more apt to be a quick and certain success. The apparatus can be worn with perfect ease, does not destroy the child's comfort in his daily play and work at school, and does not interfere with the movements of the jaws in mastication and talking. I understand that Dr. Allan has had considerable experience in this process.

*Dr. Allan.*—I am quite certain that there has been more than one apparatus described that were more or less successful. They have all adopted the same general principles. There is no bending of the jaw; it is simply forced back in its socket. There is a thick double cartilage where the lower jaw is set into its socket, which yields very readily to pressure.

*Dr. G. Evans.*—I do not ordinarily apprehend injury to the permanent set by exerting pressure on the jaw or on the temporary teeth during their presence. I believe in gentle gradual pressure in changing the arch and position of children's teeth. I am not at all in favor of the rapid work done by some.

In a practice of over thirty years I have seen several cases where central incisors have had pulps devitalized by such methods. The occlusion should always be considered in moving the teeth, as the occlusion often decides of itself the position of a tooth.

When a bandage is used to reduce protrusion of a jaw, to be effective it should be applied at an early age.

*The President.*—When does the change take place in the occlusion and in the position of the jaw?

*Dr. Allan.*—It depends upon whether pressure is kept up steadily. Some object to wearing the bandage at all times during the day, but it is not wise or safe to remove the apparatus any portion of the time, and it can be worn with as little discomfort as any other regulating appliance.

*The President.*—All this seems to lead up to the principles of regulation. I have seen the hair taken off the head and the child's mouth begin to grow out of shape before retrogression of the lower jaw was accomplished. I do not mean to assert that it cannot be done, but I should like to know from those who have practised it the principles upon which it is accomplished.

*Dr. C. B. Parker.*—I have accomplished retrogression of the lower jaw in several instances. In those cases I have used a net, like the old-fashioned nets the ladies used to wear, and I never yet had any trouble of the kind referred to. I never hesitate to take charge of a case, and I always expect success. The age I prefer is from ten to fourteen.

*The President.*—I hope that Dr. Parker will favor us later on with a full account. I for one would be glad to learn.

We will now hear the paper of the evening from Dr. Robert H. M. Dawbarn, Professor of Surgery in the New York Polyclinic Medical School and Hospital, entitled "False Tonsils."

(For Dr. Dawbarn's paper, see page 345.)

*Dr. R. H. M. Dawbarn.*—Waldeyer's tonsillar ring is composed of six lymph-glands connected by means of a chain of lymphatics.

*The President.*—I see we have the honor to have associated with us this evening two gentlemen who have been put down to open the discussion, Dr. Delavan, Professor of Rhinology in the New York Polyclinic, and Dr. Phillips, Professor of Otology in the New York Post-Graduate Medical School and Hospital, to criticise Dr. Dawbarn as thoroughly as they may.

### DISCUSSION.

*Dr. D. Bryson Delavan.*—Mr. President, It would not be possible for us to discuss all of the points which have been touched upon in the admirable paper to which we have just listened. There are a few, however, to which I would like to refer. It seems to me that the most valuable suggestion to be derived from the paper is one which has occurred to me during the reading of it, namely, that the condition of lymphoid hypertrophy, of which we have been hearing, is one of great practical interest to the dentist. There is no doubt that obstruction of the upper air-passages and consequent mouth-breathing is the occasion of a large number of varieties of deformities of the upper jaw and hard palate, and therefore the indirect cause of serious malposition of the teeth. It can be proved without question that these deformities are due to the obstructed breathing, and it can also be shown that if the child be young enough and the obstruction to respiration be thoroughly removed, commencing deformity of the hard palate and upper jaw can be checked and an improved condition in the shape of the parts secured by the restoration of normal breathing. In consequence of this it is important to recognize the fact that efforts made by the dentist to correct malpositions of the teeth are directly hindered by the results of obstructed respiration. If, therefore, the faulty position of the teeth has been due to such obstruction, it becomes evident that efforts made to straighten the teeth should not be undertaken until the original causes of the trouble have been themselves removed.

In any case where a course of treatment is required for the straightening of crooked teeth, the dentist should discover, if possible, whether the patient is a mouth-breather or not. In case mouth-breathing be present, he should see to it that the condition is corrected before he undertakes the straightening of the teeth. If this suggestion be observed, this work will be rendered much easier and the possibiliy of improvement greatly increased.

Straightening the teeth in these young subjects is a somewhat serious matter when we consider that it must be done at a period of great general constructive activity and at a time when the child is pressed with the ordinary duties of school, with outside lessons and occupations of a trying character, with more or less of the excitement of social life, and with all of the necessary unrest and discomfort which are apt to be present at this time of life. Such a child suffering from a serious obstruction of the nasal breathing is naturally delicate and nervous, and it is asking too much of him to expect that all of the above-mentioned things should be carried on at one time without serious detriment to his general health. I think, therefore, it cannot be too strongly urged that the general health of the patient be built up by the securing of normal breathing before serious work upon the teeth is commenced.

A few words as to diagnosis and treatment: While the diagnosis may be readily made by the insertion of the finger into the child's pharynx, this practice is a disagreeable one to the patient, and should never be employed unless it is impossible to demonstrate the parts by the method of posterior rhinoscopy for the examination of the upper pharynx by means of a strong light reflected into it from the surface of a little mirror. It is necessary, in order to secure the best results in examinations of these patients, to use great gentleness and skill.

With regard to the use of anæsthetics: After many years' experience, during two of which I used chloroform exclusively in upward of two hundred operations, I am ready to say, without hesitation, that I prefer ether in these cases to any other anæsthetic. Properly administered, it is not necessarily more irritating than chloroform, and the whole testimony of modern research is to the effect that it is far safer. Nitrous oxide is not so easy of administration to young children as it is to adults, and in cases of marked obstruction of the upper air-passages its results are sometimes disagreeable.

The diagnosis of lymphoid hypertrophy at the vault of the pharynx is usually easy to make. In some cases the facial expression of the child is all that is necessary to prove the presence of these growths.

In general it may be said that where the lymphoid tissue of the pharynx is increased sufficiently in size to cause obstruction, or to produce an undue amount of secretion with result of obstructing the pharynx and injuring the surrounding structures, the growths should be removed. It is not too much to say also that the operation for the removal of these growths is not entirely a simple one, and that it cannot with propriety be intrusted to inexperienced hands.

*Dr. Wendell C. Phillips.*—I suppose from the programme that possibly you might expect me to discuss this paper from the standpoint of the otologist, but I rather prepared to speak from your own, so far as my own experience would permit.

I did not hear the first part of the paper, but I think the point that you would like to have settled is, first, whether the presence of enlarged tonsils and the presence of lymphoid tissue in the vault of the pharynx are productive of deformity in the upper jaw. If I take a little different view from the writer, I mean no reflection on the paper. I have come to think that we have made a great bugbear of adenoids as a cause of deformity of the upper jaw.

I have recently examined a large number of adenoid cases, both in private and hospital practice, with special reference to deformity, and have found in many cases no tendency to maxillary deformity, especially in young children from two to ten years of age.

In the *Journal of the American Medical Association*, February 15, 1896, Dr. Eames, of Boston, says, "It has been said that the adenoid growths in the pharyngeal vault cause irregularities of the teeth. I do not believe this to be the case, but rather that the dental irregularities are only another expression of the same cause that operates to produce the adenoid growth; in other words, there is one cause common to both."

Dr. Talbot, of Chicago, believes "that there are many cases of contracted arches where mouth-breathing does not exist; there are also many cases of normal arches where it is present."

It is only in selected cases where we can be sure that the condition has been brought about by the presence of adenoid tissue in the nasopharynx. Perhaps the very severity of the case may have something to do with it. That is to say, in severe cases the high contracted arch may be developed. If this be true, the longer the adenoids are allowed to remain the more marked the contraction would become. I personally do not think that ordinary enlarged tonsils alone have much to do with the deformities of the jaw.

The point that was brought out by the writer in reference to removing the adenoids and tonsils is very practical. I think that as soon as adenoid growths are discovered it is important to follow with prompt surgical interference.

The ear is so closely associated with the pharyngeal vault that the otologist is brought to realize the results of these growths more markedly than perhaps any other people who are practising. The ears seem to be special victims to these pests of child life.

I have come to believe that the necessity of introducing the finger into the vault of the pharynx exists only in very rare cases. I have tried to think since coming in this evening of the number of times within the last three months I had used my finger in this way. If it had not been for one patient, and that this afternoon, in the hospital, I should not have recalled any, and I did that more because I was pushed for time than for any other reason. To begin with, to introduce the finger into the vault of a young child is a barbarous thing. I rarely fail to accomplish all I desire with a tongue depressor and a mirror. Once get the confidence of the child and the examination is easily made.

It is far better to see the growths than to feel them. The digital examination is so painful that the child immediately becomes one's enemy. Indeed, in many cases the diagnosis can be made without resorting to either. Children with chronic suppuration of the ears are almost always the victims of adenoids. There is a certain condition found in the drums which I have come to regard as almost pathognomonic of adenoids. I refer to the reddish-brown color of the drum so often observed.

The presence of lymphoid tissue in the vault of children is far more common than it is believed to be, and where it is present it should be removed at once. Such operation may not be required on account of any immediate danger to the nose and throat, but the danger to the ear is certainly very great, and even a small quantity of the growth in the vault is a menace to the ears. There are many cases where there will really be no difficulty in breathing. I do not think there is any special difficulty in breathing in over one-half of the cases of lymphoid tissues in the vault. Many other cases show but little. We have to find it out from something more than the mouth-breathing or snoring at night.

The Valsalvian method in testing whether the child has adenoids or not may be very good, but children with adenoids usually experience more or less trouble with their hearing, and I would not think it safe to make use of this method or test for fear of inducing thereby a suppurative inflammation of the middle ear.

We all know that the reader of the paper this evening did not speak of "thumb-suckers," to which Dr. Delavan has alluded, with any degree of seriousness, especially in speaking to dentists, who all know of the dangers of this habit.

If the hygiene in the treatment of these diseases could be commenced early enough, something might be done towards preventing in the very young child the hypertrophy of the pharyngeal tonsil. Dr. Jonathan Wright has examined the tissues involved in fœtal life, and has frequently found marked lymphoid growths.

The youngest child I ever operated upon was ten months old, a case of acute middle-ear suppuration.

In most cases it is better to operate under an anæsthetic. If the tonsils only are to be removed, it is not necessary to anæsthetize. Ether is to be preferred. One may go for some time with chloroform without accident, but finally a death occurs from it, and ether becomes the favorite.

I do not think in any way there is very much benefit in cocaine anæsthesia. The application is so apt to close its effect by contact with the mucous flow. The tonsil is not a highly organized tissue, and the pain of cutting is not great. In adenoid operations, it is important that the ether be administered by a man who is accustomed to these operations. The operator should acquire dexterity in this operation, and when so performed but a short time is required. It is not at all necessary to prolong an adenoid operation; one can be thorough and still do his work rapidly.

Every man has his choice of instrument. There is one that I have found most useful. It is what is known as Brandigee forceps. That instrument, properly introduced, removes almost the entire mass at one time; then use the curette and finally the finger-nail, but except in very young children one cannot remove much of the growth with the nail.

The President has asked how I would make a diagnosis of adenoid growths. I make it from the conditions I see present: First, facial expression; second, ear symptoms; third, history; fourth, examination as heretofore indicated.

*Dr. L. C. Leroy.*—Why not use nitrous oxide alone? I have understood that the combination of nitrous oxide and ether, or a combination of ether and chloroform, have been the cause of serious accidents. I should judge that either alone would be preferable.

*Dr. Dawbarn.*—For the simple reason that in nitrous oxide alone it is necessary to have a mask that closely covers the respiratory region. As soon as the mask is removed, or certainly with the second or third breath, the patient will be awake and struggling, which is a dangerous thing to allow during the operation. Give gas first, if that is desired, and follow by ether. I shall have to differ with Dr. Leroy in his idea that primary use of gas followed by ether or chloroform has been the cause of serious accidents; if so, I have not heard of them.

Two points are perhaps worth dwelling upon. Chloroform can with sufficient skill often be given to the child while it is asleep without awakening it, causing it to slide from the natural sleep into the artificial slumber. This cannot be done with adults, or, at least, very seldom, without awakening them. It is impossible to accomplish with ether the same result in children. An obvious advantage of using chloroform in this way is to avoid frightening nervous and easily excitable little patients. Again there is not a physican here who does not know the method used by, for instance, Dr. Bennett, of New York,—first, nitrous oxide, the child becoming quiet, then slide into either ether or chloroform anæsthesia.

*Dr. Howe.*—It seems to me that Dr. Phillips's consideration of the possibility of the lymphoid growths being the cause of the deformities of the upper arch, if I understood him, was confined to it as the only cause, while I understood the paper to consider it only as a factor; and while it is true that many cases of contracted arches have no lymphoid hypertrophy, and there are perhaps many normal arches with such hypertrophy of lymphoids, still it may be claimed that these growths often do have an effect on the maxillary bone.

It seems to me that Dr. Dawbarn's explanation of the possibility of its being produced in the way he describes is reasonable, and may account for it in a way that, so far as I am aware, has not been before suggested.

*Dr. Phillips.*—The question is, Cannot the deformity of the arch be congenital? I do not see why a contracted arch cannot be inherited as well as some other peculiar anatomical deformity. I do not know what the experience of the dentist may be in regard to it.

*Dr. Howe.*—I think the shape of the maxillary bone is as likely to be inherited as any other feature of the face. I have seen peculiar positions of the teeth—such as a peculiar twist in a lateral incisor on one side—descend through three generations, and peculiar aberrations of form and relation of jaws descend to children, so that I believe that many contracted arches are also inherited. I do not suppose that any of us believe that contracted arches are always due to the means under consideration to-night, but that they may be due to the influence of these growths in some cases has seemed to me very probable.

We are very much indebted to Dr. Dawbarn for suggesting the way in which it may take place, and to Drs. Delavan and Phillips for their discussion of the subject.

*Dr. C. O. Kimball.*—I wish to ask one or two questions. Are we to understand that this series of lymphoid structures is a normal condition found in all cases, so that this pathological condition is simply the hypertrophy of what always exists in a normal form? The second question is, In case a young person comes to us with a narrow contracted upper jaw, what other appearances would suggest the presence of these lymphoid bodies?

*Dr. Dawbarn.*—In answer to Dr. Kimball, "Waldeyer's tonsillar ring" is not a pathological condition. It is always present,—that is, as a very slight aggregation of lymphoid tissue at the points named and connected by lymph-vessels, but in most cases it would take microscopic sections to prove that the structures are always there.

Normally one ought not to feel any lymphoid tissue at all in the pharynx. The second point I thought I carefully covered,—*i.e.*, in regard to how one could detect the adenoids.

As to examining with the finger, to which Dr. Phillips alluded adversely, you will, I am sure, recollect that I preferred and named, first, the diagnosis of the pharyngeal tonsils by the aid of the little mirror. Nevertheless, it is very customary for the family doctor to use, just for an instant, simply his finger-tip for this purpose, even at the risk of some unpopularity with the little patient subsequently. The nail of the examiner should be cut down almost to the quick, of course; and I hope I may take it for granted that the finger is washed.

*Dr. Kimball.*—Where should we expect to find these troubles on the pharyngeal vault?

*Dr. Dawbarn.*—The finger-tip is introduced along the whole pharyngeal vault, which in the little child is so small that the finger almost fills it. It is Luschka's tonsil if at the top; an obstruction at the sides of the pharynx is from the tubal tonsils.

Instead of the normal, smooth, slippery mucous membrane, one recognizes instantly by the touch the soft yielding mass, irregular in contour, resembling, as has been said, the sensation of pressing on a bag of worms.

*Dr. Allan.*—The question I would like to ask is, How far is mouth-breathing responsible for these growths? Is a dentist warranted in making mouth-breathing the reason why he should take hold of the case?

I have a patient now only eight years of age. I have examined the throat as Dr. Dawbarn suggests, and feel warranted in telling the parents that the cause of the trouble is located in the pharynx. I want to know whether, if the mouth-breathing is corrected by dental interference, the trouble would be arrested? Are we justified in taking hold of these cases at an earlier age than we otherwise would, mouth-breathing being a cause, and a very decided one, of producing these growths and also tonsillar troubles? As a rule, in regulating teeth the dentist wants to wait until the child is well rid of all the first teeth, thirteen to fourteen years, but in these cases earlier interference seems necessary and proper.

In this special case I have advised the parents to commence dental treatment at once, not on account of the teeth, but because it can serve as a preventive to these troubles in the throat.

I have been very much interested in the paper read and discussed by the gentlemen who have been with us this evening. I would like to have some light on this very important point to us dentists. Are we right in instructing parents that mouth-breathing should be remedied at the earliest possible moment, and that the dentist must first be consulted?

*Dr. Dawbarn.*—I can answer that question very definitely and positively, and I am sure that Drs. Delavan and Phillips will agree with me. When the dentist meets a case of adenoids, he should in the interests of his little patient refer him at once to a surgeon. He is derelict in duty if he does not do this. Again, as to diagnosis without any direct examination by the finger or otherwise, if you find a child who has a mouth-breathing habit, and who usually snores in his sleep, such a child in an enormous majority of cases is suffering from the disease in question, and you can be reasonably sure you are all right in saying so. If he also has large faucial tonsils, this even increases the certainty of the diagnosis.

While on the subject of diagnosis I wish to emphasize the fact that dentists should not ignore abnormal conditions observed in the mouth, aside from the teeth. As members of the medical fraternity they have an added duty to their patients to perform in advising them to seek prompt help. As a case in point, some little time ago a case of leucoplakia lingualis, or "wash-leather tongue," which in twenty-five per cent. of instances ultimately ends in cancer of the tongue, was sent to me for diagnosis and treatment. The patient had to have an operation for the removal of one-half of his tongue, in order to save his life, for the disease was already well advanced. His dentist had seen his mouth a good many times; why had he not spoken to the patient about it, and advised earlier care that might have prevented the need for that operation? The patient spoke quite bitterly of his family dentist in this regard.

*Dr. S. C. G. Watkins.*—I have been much interested in this discussion. I would like to speak on one point which has not been made clear to me, and that is the effect of adenoids upon the mouth, and how the arch is affected. Several years ago our friend Dr. Delavan operated upon a case, a boy of twelve, who in infancy had a perfectly round normal mouth. There was a growth of adenoid tissue from about the fifth or sixth year up to the time the boy was twelve years of age. I made impressions of the mouth several times between the ages of seven and twelve, showing the change in the arch, it gradually becoming more narrow and pointed each time an impression was taken.

The arch ceased to grow narrower and higher after the operation. Since that time he has breathed through his nose to a considerable extent, although sleeping most of the time with his mouth open. As an effect of the adenoid growth, the child is compelled to breathe through the mouth, and the result is the buccinator muscles are drawn down and the arch is contracted by the pressure. The muscles of the lower jaw are drawn up and in, and they have a tendency to flatten it, just the opposite condition from that found in the upper jaw.

This one case I speak of because it has been under my own eye during all these years. The boy was anæmic, weak, miserable always during the presence of the adenoids. Since the operation he has grown steadily, is now nineteen years old, six feet two inches in height, and weighs one hundred and ninety-five pounds. In that case neither father nor mother had a high narrow arch. The change in the shape of the arch was constantly perceptible up to the time of the operation.

Dr. Phillips made the statement that the adenoid growths do not always cause the high arch. That may be due to the fact that in some cases the bones may be of a more dense nature than in others, and not yield to the pressure of the muscles. We all have seen cases where people never clean their teeth, and yet they resist decay, and others who die of consumption whose teeth are in perfect condition; yet we expect to find poor teeth in diseased mouths and in the mouths of those with frail constitutions; in like manner I think the rule will hold good as to the adenoid growths not always affecting the arch.

*Dr. J. Bond Littig.*—I have always had an idea, when I found a case of mouth-breathing, that there was something abnormal about the nose or throat and usually send the patient to a surgeon for treatment.

I believe there is another muscle which occupies a prominent place in the shaping of the arch,—that is, the tongue. When one breathes through the mouth the tongue rests on the lower arch but does not touch the upper, so it does not have a chance to exert its influence as it should in shaping the upper arch. This I feel sure is one of the reasons why mouth-breathers have contracted arches.

*Dr. Phillips.*—I have been glad to get some expression of opinion from your stand-point as dentists. If the child goes more than one year and has no teeth, we are told that this is a symptom of rhachitis. May not some of these forms be due to a malnutrition of the bones, which when associated with adenoids would much more easily cause such deformity? I believe there must be something else besides the adenoids in order to cause such deformity.

*Dr. Dawbarn.*—I think the reader of the paper is very fortunate in having such distinguished brothers from the outside to discuss his paper. I am very proud of the service which they have rendered me to-night.

It is very natural, in the course of reading a paper as long as mine, and reading it as rapidly as I did, that a few of the many things discussed therein should not be remembered by my hearers. I believe I alluded to the atmospheric pressure among causes leading to the partial obliteration of the useless respiratory nasal space when the pharynx is obstructed. In discussing the stigmata of degeneration I mentioned the fact that the high narrow arch is occasionally due not to degeneration but simply as a mark of inheritance.

Regarding the question of choice in anæsthesia, we cannot to-night go fully into that immense field, which only a little while ago I discussed in a pamphlet upon "Threatened Death during Anæsthesia, and its Treatment," and which was sent to all the members of this Institute.

There is absolutely no peculiarity about this operation requiring any different anæsthetic from any other operation of the same length. As to chloroform, in skilled hands it is safe, and children's specialists—Jacobi for example—teach that in children it is especially so. When the eminent Dr. von Nussbaum could bear witness, as he did, that he saw chloroform administered forty thousand times, and without a death, no better proof seems needed that deaths from chloroform mean unskilled handling, as a rule. We must also remember that the proportion of ether deaths (which Dr. Weir gives as one in two thousand in New York Hospital) would mount even higher if we included those who die weeks and months later on from the lighting up of latent kidney and lung diseases by this irritant cause. Of course, it is admitted that there are conditions in which we would prefer ether, and it is much the safer with an anæsthetic with but small experience than is chloroform.

Concerning the Valsalvian method of testing the Eustachian tube, regarding which Dr. Phillips takes issue, that is not, of course, original with Dr. Dawbarn. I, too, should oppose its use save in children whose pharynges were first cleansed by the douche. It is certainly not necessary to help the diagnosis of pharyngeal tonsillar growths. It was only named as one means whereby we can determine whether these growths have already encroached by pressure on the tubes.

Regarding the special forceps of which Dr. Phillips is so fond, while he was exhibiting them Dr. Delavan remarked, "Dangerous instruments!" This is a case in illustration of the point Dr. Phillips himself made,—namely, that the question which instrument is best to use is largely a matter of the personal equation. In Dr. Phillips's hands I do not doubt, and I am sure neither does Dr. Delavan, that the forceps in question are safe, though for most I think it would be unsafe as compared with the Gottstein curette.

I must beg to differ with the opinion expressed by Dr. Phillips that dentition delayed beyond one year means rickets as its cause. I know of cases to the contrary, although it is doubtless true that late dentition is one among a half-dozen or so of important signs of the onset of this disease.

*The President.*—This growth which we have had under discussion this evening has happened to come under my attention at various times. A dentist in Boston, a man of marked ability, invited me once to examine the irregular teeth of his son. When I saw the boy I immediately said, "Why, have you examined for adenoids?" The reply was, "I never thought of that." Here was a boy about sixteen years of age, the father giving himself to our work, yet he had never before had thought of lymphoid growths. The boy was operated upon immediately. There was found to be considerable lymphoid growth; the boy grew rapidly after its removal, the teeth were brought into their normal position, and there is nothing marked about him now, though the prognathous condition of the upper jaw was a great deformity when I first saw him. I mention it to show how such cases may come before us, and if we are not prepared to diagnose them, we may fail to render the greater service needed.

Another case, a young lady, sixteen years old, suffered from adenoids complicated with an irregularity of teeth. She had consulted a dentist, who, after moving the teeth into new positions, cut the cusps off because they interfered with closing the teeth. I could do nothing for the young lady unless these adenoid growths were removed. Upon their removal the teeth were regulated as nearly as I was able, considering that there had been certain teeth extracted, and a retaining plate was inserted. The young lady began to grow in size, strength, and beauty; health was confirmed, and in three years she was described as "a tearing beauty." A stronger, healthier woman one would not wish to see. She was married one year ago. The extraction of teeth and the loss of her cusps have interfered with the function of mastication. I can hardly say enough against the way the cusps were mowed down like grass before the sickle. It seems to me that a surgical interference should have been called for by both these dentists, in order to save the teeth and so retain complete mastication.

It would seem to me that the existence of adenoids in fœtal life goes to show that irregular teeth and narrow, high upper jaws may be congenital as well as the adenoids. That is a field in which we can study a good deal before we find out all there is in it.

We are hoping to be able to find out some time whether the irregularities in the teeth are dependent upon adenoids, or whether adenoids are the cause of irregularities in the teeth.

*Dr. Kimball.*—On behalf of the Executive Committee and the Institute, I desire to express our very deep gratitude to these three gentlemen,—extremely busy men,—who have come among us, each bringing some special point of diagnosis, pathology, or treatment, not only for the purpose of enlightening us, but to enable us in our ordinary routine work to understand the cause of certain naso-pharyngeal conditions which affect us directly, their discomforts and dangers, and the means of their removal. I move that a vote of thanks be extended to Drs. Dawbarn, Delavan, and Phillips for favoring us with their presence this evening and this interesting discussion.

Carried.

Adjourned.

FRED. L. BOGUE, M.D., D.D.S.,
*Editor The New York Institute of Stomatology.*

